# The Effect of Surgical Intervention for Delayed Cervical Central Cord Syndrome

**DOI:** 10.1155/2017/7979850

**Published:** 2017-04-09

**Authors:** Yanan Liu, Zongyi Wang, Shaofeng Yang, Huilin Yang, Jun Zou

**Affiliations:** Department of Orthopaedic Surgery, The First Affiliated Hospital of Soochow University, Suzhou, Jiangsu 215006, China

## Abstract

The authors retrospectively studied 11 patients with delayed cervical central cord syndrome (CCS) to investigate the efficacy of the surgical intervention on treatment for delayed CCS. The American Spinal Injury Association (ASIA) motor scores, Japanese Orthopedic Association (JOA) scores, SF-36 scores, and neurologic status were analyzed preoperatively and at each time point of postoperative follow-up. The results show that patients with reversible spinal cord injury caused by delayed central cord syndrome can recover significantly after surgical intervention. Therefore, we suggest that surgical intervention is still the ideal choice for delayed cervical central cord syndrome.

## 1. Introduction

Cervical central cord syndrome (CCS) is one of the most common acute incomplete cervical spinal cord injuries, which was first described by Schneider et al. in 1954 [1]. CCS is characterized by a motor weakness more severe in the upper than in the lower extremities, usually with bladder dysfunction, and variable sensory loss below the level of the lesion [1–3].

Surgical treatment for CCS was contraindicated for many years, because the natural history of CCS has been associated with a fairly good prognosis [1]. Recently, the authors of several long-term follow-up studies of patients with CCS undergoing conservative management have reported late-onset neurological deterioration, atrophy of the hand intrinsic muscle, disability of upper limb function, and so forth.

Currently, an increasing number of spinal surgeons recommended surgical treatment. Meanwhile, investigators have reported the efficacy and safety of surgical intervention for CCS [4–6]. Our previous study also supports the viewpoint that surgery could be safely performed in patients afflicted with CCS with spinal cord compression and/or cervical instability. It has been demonstrated that surgery for CCS improves the neurologic recovery and the quality of life compared with nonoperative treatment [7]. It is generally accepted that surgical treatment is necessary for central cord syndrome (CCS) with an underlying cervical stenosis. As for CCS with evidence of significant spinal cord compression, we suggest performing surgery as soon as possible. Of course, that the physical condition of the patients could tolerate the surgery is the prerequisite.

Many patients with CCS undergo delayed surgery because of different causes. However, the information about the efficacy of surgical intervention of delayed CCS is sparse. Therefore, we aimed to evaluate whether surgical treatment would be beneficial for the neurological outcome of patients with delayed CCS.

## 2. Methods

### 2.1. Patients

Between January 2005 and December 2015, 11 patients with traumatic CCS were treated at our institution. The interval time between injury and surgery was more than 30 days (mean time, 90.64 days). Of these, 11 patients (mean age, 54.18 years old, M/F = 10/1) who were followed up for more than 6 months were included in this study. All of the patients were undergoing local spinal cord compression or preoperative kyphosis. Causes of injury include traffic accident in 4 cases, injury due to falling from height in 3 cases, falls in 2 cases, and being injured by falling objects in 2 cases. Concomitant injury elsewhere in the body was observed in 3 cases. There were 10 patients with spondylotic associated changes (disc herniation or ossification of the posterior longitudinal ligament) compressing the canal but no bony damage and 1 patient with fractures and dislocations of the cervical spine. The demographic characteristics of the entire study population are outlined in Table 1.

### 2.2. Type of Surgical Treatment

The type of surgery was determined by clinical examination and images and the details of compression segments. Surgical management of CCS consists of posterior, anterior, or combined approaches, in order to achieve spinal cord decompression with or without stabilization. Four patients were operated on using an anterior approach, the primary indication being discectomy and fusion, and 6 patients had a decompressive laminectomy via a posterior approach, with or without fusion. Another patient with C2 fracture was treated by open reduction and internal fixation via a posterior approach. Routine rehabilitation exercises were recommended to postoperative patients.

### 2.3. Data Collection and Analysis

The ASIA motor scores (AMS) [8] were recorded at the time of admission (aAMS), 6 months postoperatively (6AMS), and final follow-up (fAMS). Rate of recovery of motor function was calculated as follows: (6AMS − aAMS)/(100 − aAMS) × 100% and (fAMS − aAMS)/(100 − aAMS) × 100%, recorded as 6RR and fRR, respectively. A similar approach was used in the JOA scores [9] and SF-36 scores [10].

Neurologic outcomes were compared using ASIA impairment scale. Patients are classified into classes A–E depending on their motor and sensory function according to ASIA scale [8] (Table 2).

All values are expressed as means ± standard deviation (SD). A paired *t*-test was performed to compare the admission and 6 months' postoperative ASIA and JOA scores as well as admission and final follow-up scores. Statistical comparison of ordinal ASIA impairment scale date between groups within the initial and final neurological outcomes was calculated using the Mann–Whitney* U* test. Differences were considered significant when *p* value was less than 0.05.

## 3. Results

In the study group, the average aAMS was 72.09 ± 10.79, and the average 6AMS and fAMS were 84.09 ± 10.68 and 87.27 ± 9.13, respectively. The ASIA motor scores showed significant improvements compared with the preoperative ones. This difference was statistically significant (*p* < 0.05). The rate of recovery of motor function (6RR and fRR), respectively, was 48.99% ± 25.03% and 59.57% ± 25.57%.

The mean JOA score at admission was 10.80 ± 1.87, whereas the mean 6-month and final-visit JOA scores were 15.30 ± 0.82 and 15.60 ± 1.07, respectively. A significant improvement in JOA scores was achieved during the first 6 months after surgical intervention (*p* < 0.05).

As presented in Table 3, the mean scores of the physical functioning, bodily pain, vitality, social functioning, and mental health of patients statistically improved compared with the period before the operation (*p* < 0.05). Statistical differences of the mean scores of role-physical and general healthy were not found to be insignificant between the time of admission and 6 months (*p* > 0.05). However, the mean scores of role-physical and general healthy improved significantly at the time of final-visit (*p* < 0.05).

The change of ASIA impairment scale (modified from Frankel) is showed in Table 4. The initial ASIA B grade patient improved 1 grade. Out of 6 initial C grade patients 3 improved 1 grade, 1 improved 2 grades, and 2 remained unchanged. Out of 4 initial ASIA D grade patients 3 improved 1 grade and one remained unchanged. This difference was statistically significant.

## 4. Discussion

The prevalence of traumatic spinal cord injury (SCI) worldwide is approximately 750 per million with an annual incidence that appears to be rising [11].

Central cord syndrome (CCS) is the most common form of incomplete spinal cord injury. And about 70% of all incomplete cervical spinal cord injuries are central cord lesions [4, 12].

Several scholars have demonstrated the biomechanical mechanisms of spinal cord injury in hyperextension injuries.

For a long time, the view of cervical hyperextension violence resulting in corticospinal tract injury leading to CCS has been widely accepted. Laters, Quencer [13], levi [14], and their colleagues suggested that direct lateral corticospinal tracts injury is more likely responsible for the clinical syndrome encountered with these injuries.

A delayed CCS usually occurred because of lack of timely and effective treatment. Common causes of delayed CCS are missed or delayed diagnoses and the choice of conservative treatment.

**Figure 1:**
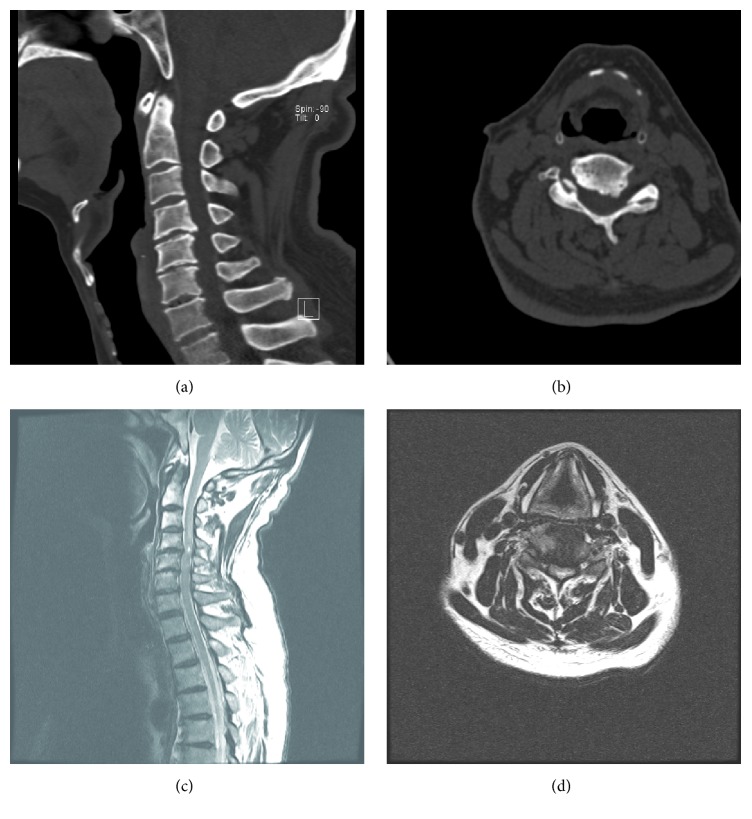
Imaging studies obtained in a 69-year-old man injured in a high falling accident. ((a) and (b)) Preoperative CT showing obvious osteophytes of C4-5. (c) and (d) MR image demonstrating C4-5 cord compression and an intramedullary high signal intensity.

The identified reasons for a missed or delayed diagnosis of CCS are as follows: (a) first reason is lack of enough understanding and alertness of CCS. In cervical injury, especially during the absence of the typical symptoms, inexperienced emergency or spinal surgeons could not make accurate judgments. At the district hospital, patients without obvious symptoms were given medication and discharged. (b) Second reason is incomplete sets of cervical spinal radiographs. Gale et al. claimed that plain radiographs were inadequate to evaluate the complete cervical spine in 72.2% of patients with blunt trauma in whom cervical spine radiographs were used to screen for cervical spine injury [15] and Gerrelts et al. reported that failure to visualize the level of injury in cervical spine radiograph was responsible for 22% of the missed diagnosis [16]. Recently, Computed Tomography (CT) and Magnetic Resonance Imaging (MRI) have provided further visualization and insight into the etiology and pathogenesis of traumatic spinal cord injuries (Figure 1). MRI may indicate the degree of traumatic injury through the presence of hyperintense signals and osseous injury as well as hemorrhage in the spinal cord parenchyma, which has been correlated with worse neurological injuries and limited recovery [17]. The presence of hyperintense signal is one of the best surgical indications [18]. (c) Third reason is painful distracting injury and poor physical examination. In addition, the CCS may be missed or delayed because of the painful distracting injury and poor examination. A patient older than 50 years with a stenotic, spondylotic cervical spinal canal incurring a hyperextension injury without evidence of fracture is a classic scenario. CCS is also seen in younger patients who experience high-velocity traumatic injuries, often with associated fracture dislocations. Some inexperienced surgeons may pay all attention to the painful distracting injury and neglect the neurological assessment unintentionally.

The choice of conservative treatment is the other identified reason for a missed or delayed diagnosis of CCS. The surgeons would recommend patients with stable or slowly improving neurologic status to take conservative treatment including cervical spinal fixation with a hard cervical collar and pharmacological interventions. These pharmacological interventions are aimed at limiting the secondary injury cascade. Some of these patients would be stabilized at an unacceptable neurological/functional level. However, some of these patients would undergo late neurologic deterioration. Patients may tend to choose conservative treatment due to religious and personal reasons. Another factor which should be considered is whether the patient has underlying diseases which have a negative effect on surgical intervention.

A retrospective study was performed in 11 patients with delayed CCS, who received operative treatment. The results of this study indicated that the reversible spinal cord injury caused by delayed CCS could obtain recovery in varying degrees. However, few patients could not achieve the desired satisfaction of overall efficacy. Poor hand function and upper limb numbness may be the main reasons troubling patients' work and quality of life, leading to the low satisfaction. We need a bigger number of cases to improve the credibility of the study. Since this study lacks a comparable nonsurgical treatment of cases and surgical treatment of old CCS cases, whether the effect was significantly better than nonsurgical treatment remains to be determined.

## 5. Conclusion

Surgical treatment can relieve spinal cord compression and improve neurological function for delayed CCS. In our opinions, we only perform surgery in the CCS cases with evidence of spinal cord compression. As for the patients without evidence of significant spinal cord compression, we recommend nonsurgical treatment including drugs, physiotherapy, lifestyle modification, and multidisciplinary rehabilitation. After surgery, the satisfaction of overall efficacy is better than the conservative treatment. For delayed CCS with the spinal cord compression, the decompression surgery is necessary, as long as the physical condition of the patients could tolerate the surgery.

## Figures and Tables

**Table 1:** Demographics of observational study cohort.

Patient	Gender	Age (years)	The interval time between injury and surgery (days)	Mechanism of injury
1	Male	63	79	Traffic accident
2	Male	58	136	Falls
3	Male	40	40	High falling
4	Male	39	158	Injured by falling objects
5	Male	69	163	High falling injury
6	Male	41	67	High falling injury
7	Male	64	31	Falls
8	Male	58	129	Injured by falling objects
9	Male	65	57	Traffic accident
10	Female	46	125	Traffic accident
11	Male	52	31	Traffic accident

**Table 2:** ASIA impairment scale.

Grade	Functional description
A	No sensory or motor function is preserved in the sacral segments S4-S5
B	Sensory but not motor function is preserved below the neurological level and includes the sacral segments S4-S5
C	Motor function is preserved below the neurological level, and more than half of key muscles below the neurological level have a muscle grade less than 3
D	Motor function is preserved below the neurological level, and at least half of key muscles below the neurological level have a muscle grade greater than or equal to 3
E	Sensory and motor function are normal

**Table 3:** Summary of admission and follow-up ASIA motor, JOA, and SF-36 scores.

Interval	mean ASIA scores	mean JOA scores	SF-36 scores							
			PF	RP	BP	GH	VT	SF	RE	MH
Admission	72.1	10.8	43 ± 7	25 ± 7	42 ± 10	36 ± 14	36 ± 10	41 ± 5	53 ± 6	41 ± 7
6 months	84.1	15.3	45 ± 8	26 ± 9	46 ± 10	38 ± 8	39 ± 8	44 ± 7	54 ± 6	45 ± 5
Final-visit	87.3	15.6	54 ± 8	34 ± 7	59 ± 9	50 ± 7	48 ± 10	47 ± 6	52 ± 5	49 ± 7

BP: bodily pain; GH: general health; MH: mental health; PF: physical functioning; RE: role-emotional; RP: role-physical; SF: social functioning; VT: vitality.

**Table 4:** The change of ASIA impairment scale.

Preoperative ASIA impairment scale	Number	Postoperative ASIA impairment scale				
		A	B	C	D	E
A	0					
B	1			1		
C	6			2	3	1
D	4				1	3
E	0					
